# Structure and microbial diversity of biofilms on different pipe materials of a model drinking water distribution systems

**DOI:** 10.1007/s11274-014-1761-6

**Published:** 2014-10-24

**Authors:** Agnieszka Rożej, Agnieszka Cydzik-Kwiatkowska, Beata Kowalska, Dariusz Kowalski

**Affiliations:** 1 Department of Water Supply and Sewage Disposal, Faculty of Environmental Engineering, Lublin University of Technology, ul. Nadbystrzycka 40B, 20-618 Lublin, Poland; 2 Department of Environmental Biotechnology, University of Warmia and Mazury in Olsztyn, Olsztyn, Poland

**Keywords:** Drinking water system, Bacterial diversity, Biofilm, Denaturing gradient gel electrophoresis (DGGE), Heterotrophic plate count (HPC), Scanning electron microscopy (SEM)

## Abstract

The experiment was conducted in three model drinking water distribution systems (DWDSs) made of unplasticized polyvinyl chloride (PVC), silane cross-linked polyethylene (PEX) and high density polyethylene (HDPE) pipes to which tap water was introduced. After 2 years of system operation, microbial communities in the DWDSs were characterized with scanning electron microscopy, heterotrophic plate count, and denaturing gradient gel electrophoresis. The most extensive biofilms were found in HDPE pipes where bacteria were either attached to mineral deposits or immersed in exopolymers. On PEX surfaces, bacteria did not form large aggregates; however, they were present in the highest number (1.24 × 10^7^ cells cm^−2^). PVC biofilm did not contain mineral deposits but was made of single cells with a high abundance of *Pseudomonas aeruginosa*, which can be harmful to human health. The members of *Proteobacteria* and *Bacteroidetes* were found in all biofilms and the water phase. *Sphingomonadales* and *Methylophilaceae* bacteria were found only in PEX samples, whereas *Geothrix fermentans*, which can reduce Fe(III), were identified only in PEX biofilm. The DNA sequences closely related to the members of *Alphaproteobacteria* were the most characteristic and intense amplicons detected in the HDPE biofilm.

## Introduction

In recent years, there has been a great increase in the use of plastic pipes in drinking water distribution systems (DWDSs) and household installations because they are easy to cut, install, and they are resistant to corrosion. Additionally, smooth surfaces of plastic pipes facilitate the removal of deposits accumulated on their inner parts. However, Rogers et al. (1994) have reported that plastic materials are easily colonized by *Legionella pneumophila.*

Microorganisms settle on the inner surfaces of pipes and form biofilm, which becomes the source of secondary microbial contamination of water. The biofilm development depends on the chemical and biological stability of water, its temperature (Bai et al. 2010), biocides concentrations (Norwood and Gilmour 2000), hydraulic conditions and stagnation periods (Melo and Vieira 1999; Lehtola et al. 2006; Rochex et al. 2008) as well as the type of material from which the pipe was made (Jang et al. 2011; Morvay et al. 2011). The quality of water passing through plastic pipes can also be worsened by organic compounds such as additives (e.g. stabilizers, antioxidants, softeners, colouring agents), monomers or products of polymer degradation (Brocca et al. 2002; Koch 2004; Skjevrak et al. 2005; Kowalska et al. 2011) migrated from plastics. These substances may be used by bacteria to support their growth in oligotrophic conditions.

Bacteria found in water supply systems are members of complex, multi-species communities. In recent years, many culture-independent molecular methods such as in situ bacterial 16S rRNA gene profiles (Burtscher et al. 2009), flow-cytometric total cell count or adenosine tri-phosphate (ATP) analysis (Berney et al. 2008; Hammes et al. 2008) have been used to study the presence of microorganisms in drinking water. Nonetheless, heterotrophic plate count (HPC) and selective plating for pathogens are still commonly used to monitor the bacterial presence in drinking water, for instance, by state sanitary inspectorates and by the water industry on the basis of existing legislation (Polish Decree of Minister of Health from 29 March 2007 on the quality of water intended for human consumption, Polish Standard PN-EN ISO 6222:2004). Among the heterotrophic bacteria in drinking water systems the pathogenic bacteria or at least opportunistic pathogens often appear.

Enteropathogenic *Escherichia coli* or other members of *Enterobacteriaceae* may appear in water supply systems due to contamination as a result of flooding, water supply failure or insufficient disinfection, Cultivated mycobacteria were detected in the most water samples taken directly from the tap in Netherlands, other Western European countries and in US (Van der Wielen et al. 2014). Other opportunistic bacteria as *Pseudomonas aeruginosa*, *Aeromonas* sp., *Burkholderia*, sp., *Stenotrophomonas maltophila* or pathogenic strains of *Legionella* sp. quite often were detected in DWDSs (Grabińska-Łoniewska 2005). A variety of methods are available for investigating biofilm morphology. In this study a direct SEM analysis of an inner surface of pipes was used to quantify mature biofilm after 2 years of the pilot installation operation.

The aim of the present study was to investigate the potential for the biofilm formation and the diversity of microbial consortia on three materials commonly used for pipe production, namely unplasticized polyvinyl chloride (PVC), silane cross-linked polyethylene (PEX) and high density polyethylene (HDPE), in operational conditions simulating the household installations. The biofilm formation potential was evaluated with scanning electron microscopy analysis (SEM) and HPC. The diversity of microbial communities in biofilms and the water phase in studied water installation was determined by denaturing gradient gel electrophoresis (DGGE) of 16S rDNA.

The originality of this work consists the studies of biofilm on different plastic pipe materials after long 2-years experiment and the system was operated similarly to real conditions with stagnation periods. The structure of biofilms were studied on fragments of pipes which were cut off from installation, not on the exchangeable coupons to mitigate influence of additional (i.e. sealing) material and to avoid placing coupons in the center of pipe interfering the flow conditions.

In the study, in depth molecular, biochemical, and microscopic characteristics of microbial biofilms in DWDSs was performed depending on the type of plastics used. The results can be used as a guideline to select the materials for network and plumbing designing.

## Materials and methods

### Experiment set-up and organization

**Fig. 1:**
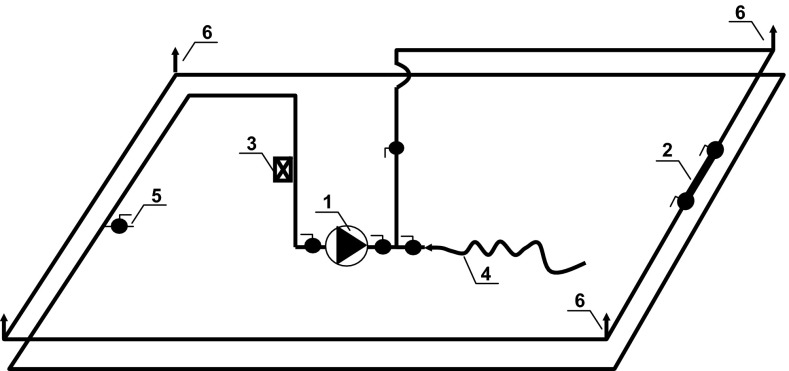
A schematic of a model drinking water distribution system; *1* Wilo pump MVIE 204-1/16/E/3-2-26 with a frequency converter, *2* removable section of the pipe for microbiological sampling, *3* ultrasonic flowmeter FD 610 (OMEGA, USA, accuracy of ±2 %), *4* flexible connection to the building water installation, *5* sampling point for water quality assessments, *6* deaerators

The pilot model water installation consisted of three independent, closed systems made of PVC, PEX and HDPE pipes; each of length 80 m and a nominal diameter of 32 mm (PVC) or 25 mm (HDPE, PEX). The installations were equipped with a circulating pumps with a variable speed frequency controllers and were supplied with tap water from the water installation (Fig. 1). The system was operated at room temperature (22 °C) similarly to real conditions when the thermal isolation of household installation was insufficient.

The tap water (100 % treated, chlorinated ground water) used in these studies had the following characteristics: pH 7.11, alkalinity 6.45 mval L^−1^, hardness 7.6 mval L^−1^, NH_4_^+^–N 0.05 mg L^−1^, NO_3_^−^–N 0.2 mg L^−1^, soluble Fe 0.7 mg L^−1^, Mn 0.01 mg L^−1^, $\hbox{PO}_{4}^{-3}$ 0.12 mg L^−1^, free chlorine 0.2 mg L^−1^, total ATP concentration 0.07 pg mL^−1^, TOC 0.2 mg L^−1^ and a HPC of 63 CFU mL^−1^ at 22 °C. During the operation period water was alternately recycled for 12 h through the model systems at a speed of 0.2 m/s, then left in stagnation for 12 h. The water consumption in household installations and specially in public buildings is irregular and concerns a night stagnation of water. The research conducted by the authors showed that in Polish conditions the vast majority of pipelines operated under oversizing. Very often maximum flow velocity distribution lines do not exceed 0.2 m/s, and at night, the speed falls below 0.01 m/s. In the laboratory installations the water flow velocity was measured using the ultrasound non-invasive flowmeter (PORTAFLOW 300), than the threshold sensitivity was 0.01 m/s (Kowalski 2009b). The Reynolds number, which is used to characterize flow regimes, ranged from 3,500 (PVC and PEX pipes) to 4,500 (PE pipes)—it is transition zone between laminar and turbulent flow.

Water was completely exchanged every week. Water exchange was associated with the flushing of the pipeline for a period of 10 min at a speed of 2 m/s. Single water exchange takes 40 s, therefore, for 10 min followed 15 exchanges of water in the laboratory system. This reflects a situation in which the distribution pipes in the end parts of the water supply systems are automatically flushed temporary opening the free outflow valves.

The research conducted by the authors showed that the age of water (the retention time) in examined water supply networks ranges from 6 to 8 days (Kowalski 2009a).

### Sampling and bacterial counts

After 2 years of operation of model systems samples for biofilm analyses were taken. Before biofilm sampling, both ends of each 30-cm piece of plastic pipe containing water (no. 2 in Fig. 1) were closed with ball valves. Water samples from pipes were collected in the sterile glass vials for analysis. Biofilms were removed from pipes by 5 min shaking with sterile 425–600 µm glass beads (Sigma-Aldrich, Germany) in 30 mL of sterile water. The obtained suspension of scraped deposits was used for HPC and PCR-DGGE analysis.

HPC was determined by a spread plating method according to Polish Standard (2004) PN-EN ISO 6222:2004, which is a commonly used procedure in drinking water studies and regarded as more sensitive than pour plating (Reasoner 2004; Lehtola et al. 2007). The water samples and their decimal dilutions were spread on Petri dishes with enrichment agar medium (R2A). The plates were incubated at 22 °C for 72 h, and another set of plates at 37 °C for 48 h; and then bacterial colonies were counted. These thermal conditions of incubations are required according to Polish Standard (2004) PN-EN ISO 6222:2004. All measurements were performed in triplicate. Colonies were passaged on fresh, sterile R2A agar medium. Bacterial isolates were characterized by Gram staining and biochemical tests: API^®^20NE and API^®^ Staph (bioMerieux, France).

### SEM microscopy of biofilms

After 2 years of operation of the pilot scale distribution systems, five small pieces (1 cm^2^ coupons) were cut out from the HDPE, PEX and PVC pipes for microscopic analysis of the biofilm structure on their inner surface. The plastic coupons were protected against drying by a parafilm frame, which was filled with water from the test system. Such coupons were placed on Petri dishes on moist filter paper and stored at 4 °C for no longer than a couple of hours before microscopic analysis was performed. For the analysis a LEO1430VP scanning electron microscope with an EDX detector (Roentec, Germany) was used. The biofilm preparations were dried in a vacuum evaporator and sprayed with chromium in a POLARON SC7620 sputter coater (Quorum Technologies, England). Five randomly selected microscopic fields (627 μm^2^) on the surface of each plastic coupon were analyzed for the biofilm structure and the number of bacterial cells. On HDPE, the number of bacterial cells was counted as a sum of bacteria visible on the surface and immersed in the biofilm. Elemental analysis of deposits was performed as well (data not presented). This meant that for each type of plastic, 25 independent measurements were performed. The mean values, standard deviation and correlation coefficients were determined. The relationships between number of bacterial cells on plastic coupons was analyzed for variance by the Fischer-Snedecor test and for mean values by the Student’s *t* test or the Cochran–Cox test at the 0.05 level of probability (Zgirski and Gondko 2010).

### PCR-DGGE analysis of bacterial communities in biofilm

The biomass from the 200 mL water samples as well as 30 mL of suspension of biofilms scrapped from inner surfaces of plastic pipes were concentrated to a volume of 200 μL by a LABAQUA Kit (LABAQUA SA, Spain) by membrane filtration through 0.4 μm pore size polycarbonate filters and centrifugation in a concentrator vial. DNA was isolated from the concentrated bacterial suspensions and scraped biofilms using GenElute™ Bacterial Genomic DNA Kit (Sigma-Aldrich, Germany) according to the producer protocol including lysozyme digestion. The presence of DNA was confirmed by agarose electrophoresis in 1.0 % gel containing ethidium bromide. The DNA concentration was also measured spectrophotometrically using a Biophotometer (Eppendorf, Germany).

For PCR-DGGE, primers EUBf933 (5′-GCACAAGCGGTGGAGCATGTGG-3′) with 5′-GC-clamp and EUBr1387 (5′-GCCCGGGAACGTATTCACCG-3′) targeting the bacterial 16S rDNA hypervariable regions V6, V7 and V8 were used (Iwamoto et al. 2000). PCR mixture contained according to the producer protocol 0.2 ng/µL of target DNA, 25 μL of AmpliTaq Gold^®^ PCR Master Mix (Applied Biosystems, USA), 1.25 μL of each primer (25 pmol) and 12.5 μL of DNA-free water. Hot-start PCR was performed at 95 °C for 10 min, followed by 40 cycles of denaturation at 94 °C for 1 min, annealing and elongation at 72 °C for 1 min. During annealing, the initial temperature was 65 °C and decreased by 1.0 °C in each cycle until it was 55 °C (a “touch-down” PCR). The final extension step was at 71 °C for 7 min (modified Wu et al. 2006). PCR products (450 bp) were detected by electrophoresis in 1.0 % agarose gel containing ethidium bromide.

DGGE analysis of PCR products was performed with a D-CODE Universal Mutation System (Bio-Rad, USA) and the gels were documented as it was described by Cydzik-Kwiatkowska et al. (2012). DGGE gels were run at 60 °C and 100 V for 5 h and stained with SYBR^®^Gold (Molecular Probes, USA). DGGE bands with a high intensity were cut out from the gel, flushed with 100 μL of sterile water, suspended in 40 μL of distilled water and left at 4 °C for 24 h. DNA released from the gel was reamplified. PCR products were purified with Roti-PCR Clean (Roth, Germany) and sequenced using Sanger method (GENOMED, Poland). The obtained partial 16S rDNA sequences were compared with the sequences from the GenBank database using the Basic Local Alignment Search Tool (BLAST) and deposited in the GenBank under accession numbers KF752457-KF752475.

Similarities between the DGGE patterns were determined using the Dice coefficient (Nei and Li 1979) in accordance with the following equation:

$$\mathop C\nolimits_{xy} = 1 - \frac{2xy}{x + y}$$

where xy is the number of bands present both in samples X and Y, x = the total number of bands in sample X, y = the total number of bands in sample Y.

 This index ranges from 0 (no common bands) to 1 (100 % similarity of band patterns).

The structural diversity of the microbial communities was expressed by the Shannon-Wiener index (H) (Fromin et al. 2002) in accordance with the following equation:

$$H = - \sum \left( {n_{i} /N} \right) \;{ \log } \;\left( {n_{i} /N} \right),$$

where n_i_ is the intensity of band *i* and N the sum of all intensities of all bands on one gel track.

Łagód et al. (2007) showed that for complete characterization of a community, not only H values but also other diversity indices should be calculated. The Evenness index (V) was calculated according to the following formula (Hurlbert 1971; Magurran 1988):

$$V = \frac{H}{{H_{MAX} }},$$

where H_MAX_ is the virtual maximum value for hypothetical community when all species are equally abundant.

The value of H_MAX_ was calculated according to the following formula (Hurlbert 1971):

$$H_{MAX} = { \log }\,i,$$

where *i* is a number of bands on one gel track.

## Results

### SEM characteristics of pipe surface

An electron microscopic analysis showed the differences, both in the amount as well as the structure of the deposits (Table 1), between the biofilms formed on the inner surfaces of three types of the pipes.

**Table 1:** Characteristics of deposits on the inner surface of the pipes

	HDPE	PEX	PVC
Percentage of surface covered by mineral deposits (%)	47.52	21.64	0
Size of mineral deposits (µm^2^)			
Mean value	3.82	0.08	0
Median	0.19	0.03	0
Standard deviation	26.40	0.24	0
Total number of bacteria (cells cm^−2^)			
Mean value	1.59 × 10^6^	1.24 × 10^7^	1.59 × 10^5^
Standard deviation	9.30 × 10^5^	7.94 × 10^6^	1.59 × 10^5^

**Fig. 2:**
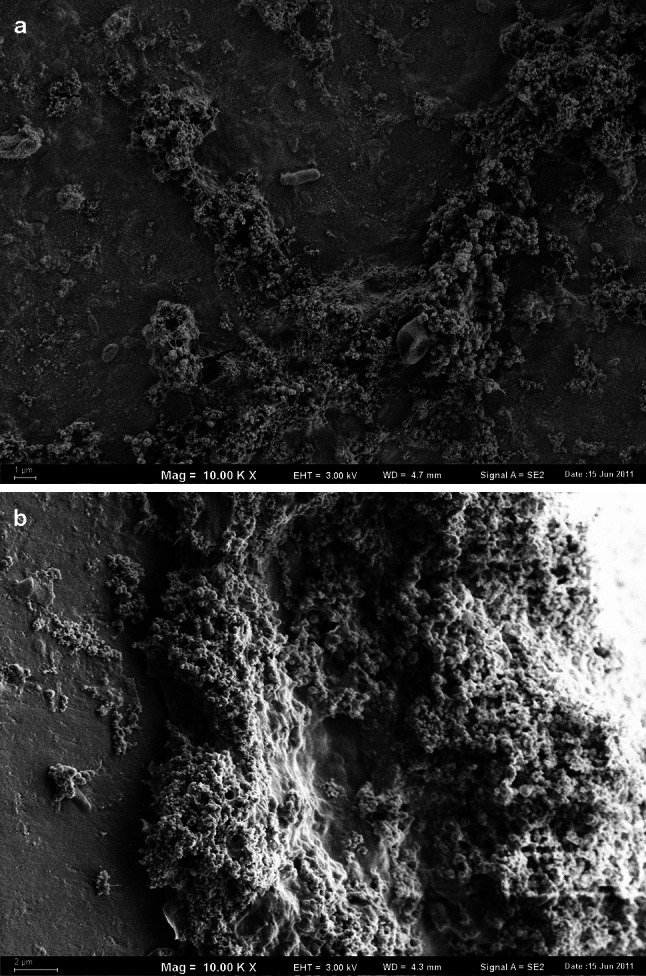
The spatial structure of deposits on the inner surface of the HDPE pipes (**a** spatial distribution, **b** vertical section, SEM, ×10,000 magnified)

The observation of the surfaces of HDPE pipes in SEM showed that they were completely covered with biofilm. Numerous recesses were present on the inner surface of the HDPE pipes, in which rust colored deposits were accumulated. The mineral deposits of irregular shape covered 47.52 % of the analyzed area (Fig. 2a). The size of the individual deposits ranged from 0.01 to 233.4 μm^2^ (n = 78, mean value 3.82 μm^2^, median 0.19 µm^2^). Their thickness reached 11.56 μm (Fig. 2b) and was substantially higher than the thickness of the deposits formed on the surfaces of the other plastics. In the mineral deposits dominated the iron oxides. The deposits were rusty-brown, and it was observed both in the light microscope and without optical devices. On the same area, the accumulations of Ca, P, S, Si, Zn and Cu were detected. Mineral compounds presumably originated from a municipal water distribution system that contained some sections of old iron pipes, and from an in-building water installation made of galvanized steel.

**Fig. 3:**
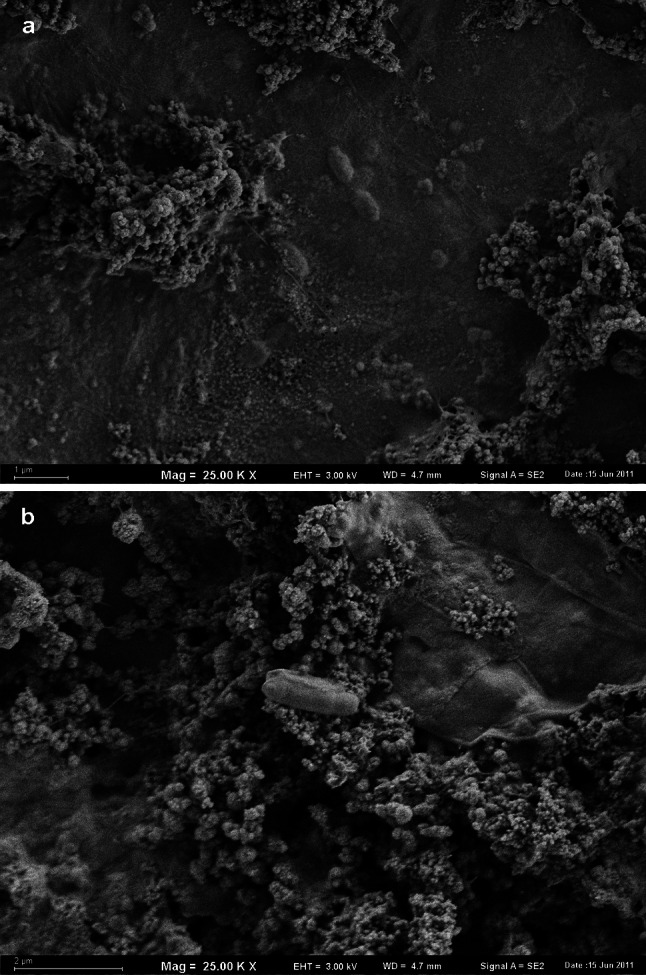
Bacterial cells in the biofilm on the HDPE surface (**a** immersed in exopolymers, **b** attached to mineral deposits, SEM, ×25,000 magnified)

Numerous individual bacterial cells of a different morphology immersed in an exopolymer matrix of biofilm on HDPE surface of pipe were observed (Fig. 3a). In the studied area of biofilm the average number of bacteria reached 1.6 × 10^6^ cells cm^−2^ (Table 1). The diameter of the bacterial cells observed in the biofilms varied from 0.32 to 0.36 µm; their length was about 0.8 µm. The diameter of coccal forms, which dominated in all analyzed areas of the biofilm on the surface of HDPE, ranged from 0.48 to 0.6 µm. Cylindrical forms of microorganisms with the diameter and the length of 0.64 and 1.83 µm, respectively, were observed more frequently on the surface of mineral deposits (Fig. 3b).

**Fig. 4:**
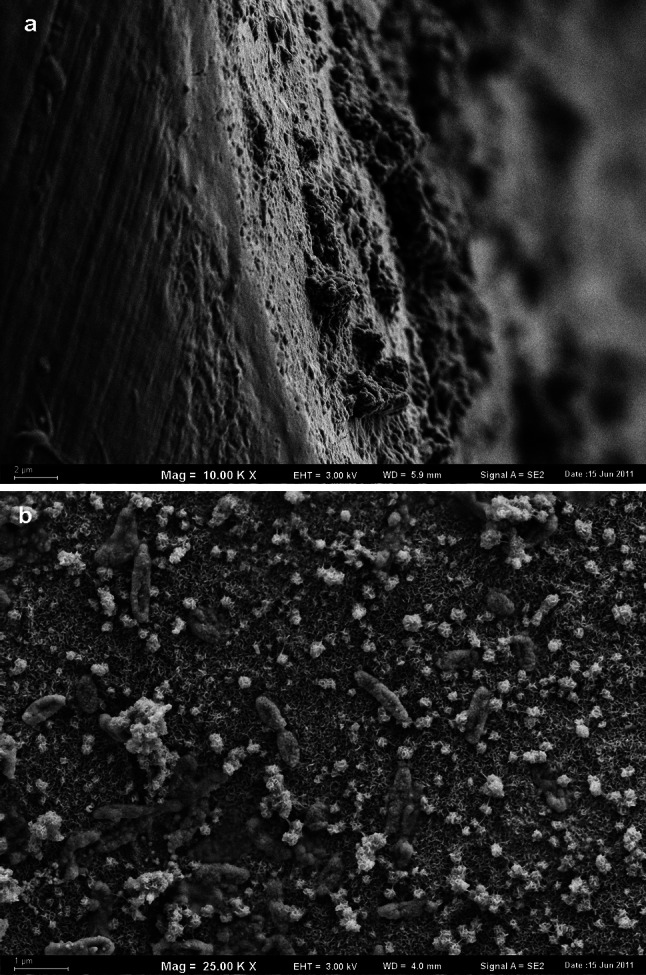
**a** The spatial structure of sediments on the inner surface of the PEX pipe (vertical section, SEM, ×10,000 magnified). **b** The spatial structure of sediments and bacteria adsorbed on the inner surface of the PEX pipe (spatial distribution, ×25,000 magnified)

The surface of PEX pipes was covered in 21.64 % by the numerous, small-sized mineral deposits (Table 1). The area of deposits ranged from 0.002 to 3.2 µm^2^ (n = 272, mean value 0.08 µm^2^, median 0.03 µm^2^). The thickness of the sediments reached up to 2.3 µm (Fig. 4a). Individual, mostly cylindrical bacteria were observed directly on the surface of the PEX pipes. Their concentration was 1.24 × 10^7^ cells cm^−2^. Bacteria did not form aggregates or colonies, and their occurrence on the surface was not associated with the presence of mineral deposits (Fig. 4b). No exopolymer layer was observed.

**Fig. 5:**
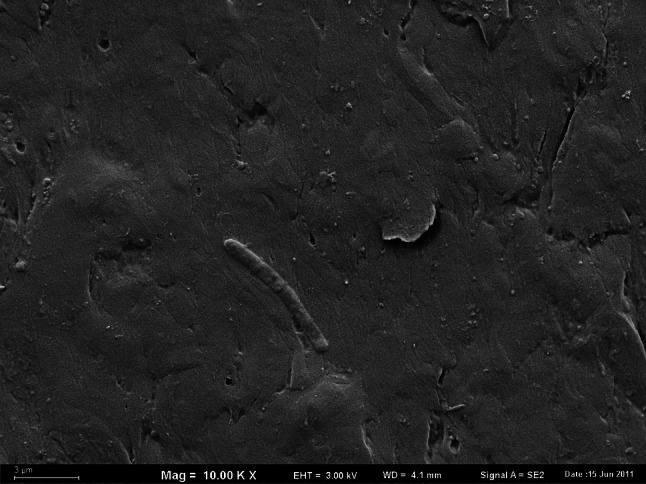
Cylindrical forms of bacteria on the inner surface of the PVC pipe (SEM, ×10,000 magnified)

The total number of bacterial cells in biofilms, estimated on the basis of microscopic analysis, was significantly lower on PVC than on other pipes. On the PVC surface, only individual bacterial cells were found, and no mineral deposits or biofilms were observed (Fig. 5).

### Cultivable bacteria in biofilm

**Fig. 6:**
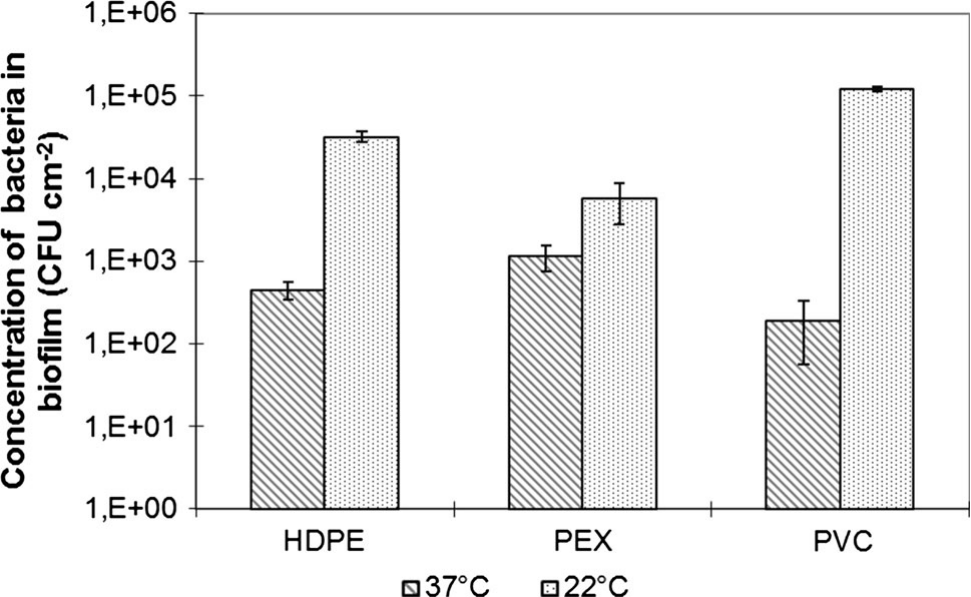
The heterotrophic bacteria plate count (HPC) in 22 and 37 °C biofilm from the HDPE, PEX and PVC pipes (mean values and standard deviation are given)

A wide range of HPC results were observed in samples of biofilm scraped from the inner surface of HDPE, PEX and PVC pipes (Fig. 6). The highest number of bacteria growing at 37 °C (1.15 × 10^3^ CFU cm^−2^) was found in the biofilm from PEX pipe. In samples from HDPE the number of bacteria was more than two times lower (451 CFU cm^−2^), but this difference was not significant. Only the number of bacteria on the PVC surface was significantly lower (193 CFU cm^−2^) than on PEX.

Significant differences in the number of heterotrophic bacteria growing at 22 °C were detected between all three plastics. The highest HPC at 22 °C, i.e. 1.22 × 10^5^ cm^−2^ was found in biofilm from PVC. HPCs at 22 °C for HDPE and PEX pipes were 3.25 × 10^4^ and 5.8 × 10^3^ CFU cm^−2^, respectively. In all three types of pipe the number of heterotrophic bacteria colonies grown at 22 °C was higher, due to the possibility of growth of both mesophilic and psychrofilic bacteria than the HPC at 37 °C, in a range of tolerance for mesophilic bacteria.

### Identification of heterotrophic bacteria in biofilm

During HPC analysis of biofilms from PVC and PEX pipes about 10 % of colonies were identified as *S. maltophila*. These bacteria dominated on the plates incubated at 37 °C. Other heterotrophic bacteria grown on nutrient agar at 22 °C formed small (below 1 mm in diameter) transparent colonies. They were rod-shaped, Gram-negative bacteria. Their passages on new agar plates, as well as an identification by biochemical microtests, failed. In the biofilm from HDPE pipes about 50 % of HPC grown at 22 °C were small transparent unidentified colonies. The rest of HPC were recognized as *P. aeruginosa* strains. Almost 100 % of colonies grown at 37 °C were identified as *P. aeruginosa*. At both temperatures (22 and 37 °C), single colonies of *Micrococcus* and *Acinetobacter* spp. were detected in all samples.

The identified strains were opportunistic bacteria that can be hazardous to health, particularly for people with weakened immune systems. The abundance of these microorganisms in the pilot installation shows their high ability to settle and multiply on the surface of the plastic pipes.

### PCR-DGGE analysis of bacterial communities in biofilm

**Fig. 7:**
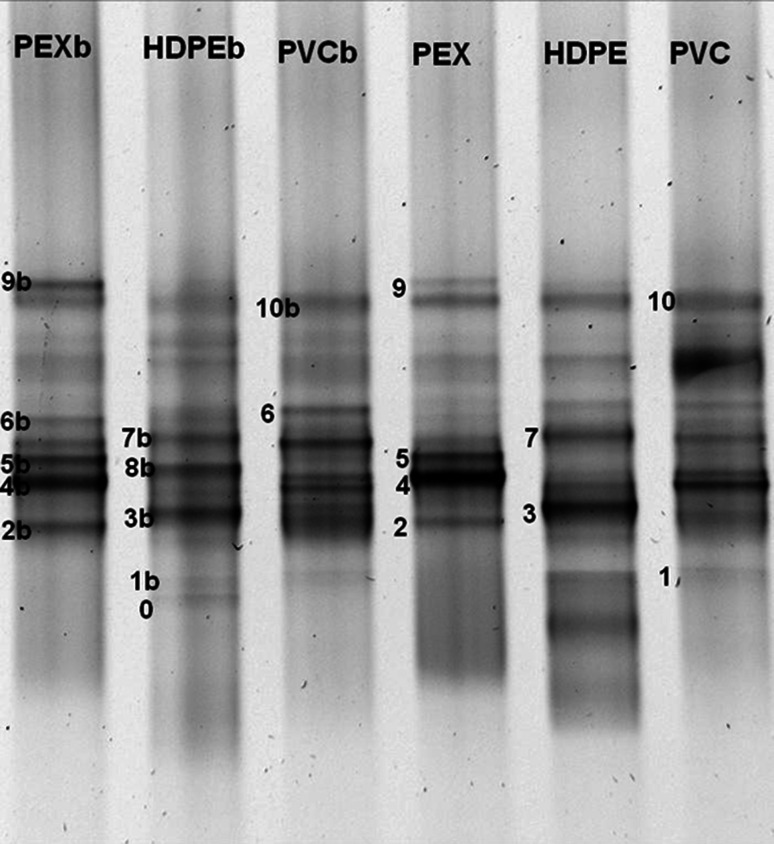
PCR-DGGE analysis of 16S rDNA from biofilm samples (PEXb, HDPEb, PVCb) and the water phase samples (PEX, HDPE, PVC) from plastic pipes

The results of PCR-DGGE are shown in Fig. 7. The Dice coefficients (C_xy_) describing similarities of DGGE patterns are presented in Table 2. The DGGE fingerprints characterizing biofilm and bacterial suspension in water phase from the pipes made of a particular polymer were very similar (>78 % similarity) but not identical. They varied in the presence and the intensity of some bands. Microbial communities in PEX and PVC pipes were more similar to each other than to HDPE samples. The highest distance was observed between the communities developed in two kinds of polyethylene pipes.

**Table 2:** The similarity of DGGE patterns for the biofilms and the water samples from different types of pipes (the dice coefficient)

Sample	Biofilm			Water phase		
	PEXb	HDPEb	PVCb	PEX	HDPE	PVC
PEXb	0					
HDPEb	0.5	0				
PVCb	0.74	0.63	0			
PEX	0.89	0.33	0.71	0		
HDPE	0.23	0.78	0.59	0.38	0	
PVC	0.74	0.63	0.89	0.71	0.59	0

A diversity of bacterial communities in biofilm and water was expressed by the Shannon–Wiener index (H). Bacterial communities in the biofilms on different polymers had similar values of H index: 2.22 (PEX), 2.20 (HDPE), and 2.10 (PVC). The H values for bacterial communities in water phase from three various pipes were lower than the values calculated for biofilms i.e.: 1.99 (PEX), 1.96 (HDPE), and 2.04 (PVC). The Evenness (V) indices for biofilms in three pipes were similar: 0.97 (PEX), 0.96 (HDPE) and 0.96 (PVC) and slightly lower in water samples to 0.96, 0.94 and 0.93, respectively.

Sequencing of bands in DGGE patterns characterizing the biofilm and the water phase biomass from different pipes showed that the type of polymer affected the composition of bacterial communities in water distribution systems (Fig. 7). The members of *Proteobacteria* (*Alphaproteobacteria, Betaproteobacteria, Gammaproteobacteria, Deltaproteobacteria*) and *Bacteroidetes* (bands No. 9 and 10) were identified in both the water and the biofilm samples from all pipes.. The most characteristic and intense amplicon in the biofilm and the water sample from HDPE was band NO. 3 (Table 3). Its DNA showed 99 % sequence similarity with DNA of uncultured *Desulfuromonadales* bacterium partial 16S rRNA gene (AM935739.1) belonging to *Deltaproteobacteria*. *Rhodobacter blasticus* (band No. 8b, 98 % of sequence similarity) and *Woodsholea maritima* (band No. 3b, 97 % similarity) belonging to *Alphaproteobacteria* were observed only in the biofilm from HDPE pipes (Table 3).

**Table 3:** Similarity of sequences of 16S rDNA DGGE bands determined by BLAST nucleotide search

DGGE bands	Closest database match of cultured taxa (accession)	Similarity (%)	Taxonomic position (lower systematic taxa)	Closest database match (accession)	Similarity (%)
0	*Acidisphaera* sp. (AB669479.1)	88	*Alphaproteobacteria* *(Acetobacteriaceae)*	Uncultured bacterium clone FH-2-29 16S ribosomal RNA gene, partial sequence (GQ162031.1)	91
1	*Pseudomonas syringae* (AJ534944.1)	76	*Gammaproteobacteria* *(Pseudomonadales)*	Uncultured *Desulfomicrobium* sp. clone GE7GXPU01B1Z87 16S ribosomal RNA gene, partial sequence (HM508007.1)	76
2	*Sphingopyxis* sp. (HM224464.1)	92	*Alphaproteobacteria* *(Sphingomonadales)*	Uncultured *Novosphingobium* sp. clone fjc-77 16S ribosomal RNA gene, partial sequence (JQ278820.1)	94
2b	*Sphingopyxis* sp. (HM224464.1)	94	*Alphaproteobacteria* *(Sphingomonadales)*	Uncultured *Novosphingobium* sp. clone fjc-77 16S ribosomal RNA gene, partial sequence (JQ278820.1)	96
3	Bacterium enrichment culture clone B30 (2011) 16S ribosomal RNA gene, partial sequence (JF830203.1)	96	*Deltaproteobacteria*	Uncultured Desulfuromonadales bacterium partial 16S rRNA gene, clone AMBD2 (AM935739.1)	99
3b	*Woodsholea maritima* (FM886859.2)	97	*Alphaproteobacteria* *(Rhodobacteriaceae)*	Uncultured alphaproteobacterium clone W-LFP137 16S ribosomal RNA gene, partial sequence (JF516154.1)	99
4	*Methylotenera mobilis* (CP001672.1)	99	*Betaproteobacteria* *(Methylophilaceae)*	Uncultured *Methylophilaceae* bacterium clone LYH1-18 16S ribosomal RNA gene, partial sequence (JQ994354.1)	99
4b	*Methylophilus* sp. (EU375653.1)	93	*Betaproteobacteria* *(Methylophilaceae)*	Uncultured bacterium clone P_21 16S ribosomal RNA gene, partial sequence (JQ810586.1)	93
5	*Acidobacterium* sp. (FN689719.1)	93	*Deltaproteobacteria (Acidobacteria)*	Uncultured bacterium clone EMIRGE_OTU_s2b2b_10774 16S ribosomal RNA gene, partial sequence (JX222537.1)	96
5b	*Acidobacterium* sp. (FN689719.1)	96	*Deltaproteobacteria (Acidobacteria)*	Uncultured bacterium clone EMIRGE_OTU_s2b2b_10774 16S ribosomal RNA gene, partial sequence (JX222537.1)	96
6	*Rhodovulum* sp. (EU642859.1)	93	*Alphaproteobacteria* *(Rhodobacteriaceae)*	Uncultured bacterium clone a-19 16S ribosomal RNA gene, partial sequence (JX040364.1)	94
6b	*Geothrix fermentans* (NR036779.1)	94	*Deltaproteobacteria (Acidobacteria)*	Uncultured bacterium clone EMIRGE_OTU_s2b2b_3195 16S ribosomal RNA gene, partial sequence (JX222379.1)	94
7				Uncultured bacterium clone a-132 16S ribosomal RNA gene, partial sequence (JX040403.1)	92
7b				Uncultured bacterium clone a-132 16S ribosomal RNA gene, partial sequence (JX040403.1)	98
8b	*Rhodobacter blasticus* (NR043735.1)	98	*Alphaproteobacteria* *(Rhodobacteriaceae)*	uncultured bacterium partial 16S rRNA gene, clone K15.94 AW (HE576390.1)	99
9	*Lacibacter cauensis* (AB682227.1)	97	*Bacteroidetes*	Uncultured *Bacteroidetes* bacterium clone VSL5W1u13 16S ribosomal RNA gene, partial sequence (EU633854.1)	99
9b	*Sporocytophaga* sp. (DQ186971.1)	87	*Bacteroidetes*	Uncultured *Bacteroidetes* bacterium clone cher4_1B_19 small subunit ribosomal RNA gene, partial sequence (JN020226.1)	88
10	*Adhaeribacter aerolatus* (HM156145.1)	87	*Bacteroidetes*	*Adhaeribacter aerolatus* (HM156145.1)	87
10b	*Sporocytophaga* sp. (DQ186971.1)	88	*Bacteroidetes*	Uncultured bacterium clone B116 16S ribosomal RNA gene, partial sequence (JF429098.1)	89

Band No. 6b was present in the PEX biofilm but was not found in the PEX water sample. The sequence analysis showed that the band was probably derived from *Geothrix fermentans* that is able to reduce Fe(III). The most intensive DGGE bands characterized the water samples from the PEX lines corresponded to the DNA sequences of microorganisms belonging to *Alphaproteobacteria* (*Sphingomonadales*) (band No. 2), *Betaproteobacteria* (*Methylophilaceae*) (band No. 4) and *Deltaproteobacteria* (*Acidobacteria*) (band No. 5). These DNA bands were not detected in the HDPE lines.

## Discussion

Despite the well-known fact that only small fraction (in many cases <1 %) of microbial communities in water and biofilm can be cultivated on conventional media (Van der Kooij et al. 1995; Hammes et al. 2008) the HPC and selective plating is still important method for detection of pathogens and opportunistic heterotrophic microorganisms.

In the present study, the number of bacteria estimated by HPC studies and SEM observation between biofilms from the HDPE, PEX and PVC pipes differed. The described discrepancies in the number of bacteria may be a result of species differences in populations in three biofilms, the presence of uncultured bacteria, as well as differences in the structure of the biofilm. During the mechanical removal of the biofilm from the surface of the pipes, the large parts of the mature biofilm could be scrapped from the HDPE pipes and spread on agar plates as inoculum. Finally, colony counts were probably underestimated. A small amount of extracellular polymers may favor the release of smaller fragments or even single bacterial cells, what may reduce the disparities between the total number of bacteria by SEM and the HPC.

Rogers et al. (1994) reported that *L. pneumophila* easily colonize plastic materials. They arranged tested materials with respect to biofilm formation intensity in ascending order: glass < stainless steel < PP < PVC < mild carbon steel < PE < Latex. Different polymers can vary in an affinity to surfaces of bacteria (smoothness, electrical charge, Zeta potential, abundance of active chemical groups) influencing on first step of colonization as adhesion (Triandafillu et al. 2003). Plastic pipes can release a variety of chemical compounds (monomers, low molecular weight polymer units and additives as well as degradation products). The pollutants migrated from HDPE pipes were identified in numerous studies as phenols, quinones, aromatic hydrocarbons, aldehydes, ketones, esters, terpenoids and some of those compounds may be used by bacteria as carbon source degradation (Brocca et al. 2002; Koch 2004; Skjevrak et al. 2005; Kowalska et al. 2011). PVC pipes release various chlorinated compounds, organotin compounds as well as VOC, mainly aldehydes (Kowalska et al. 2011) and these compounds can be more toxic for bacteria then substances released from other pipes.

Yu et al. (2010) also reported the differences between a biofilm formation potentials of different materials. On the basis of total ATP concentration and *E. coli* number, authors evaluated a higher biofilm formation potential of polyethylene than PVC coupons. The microscopic analysis conducted in the present research also showed the difference in the amounts of deposits on the surface of different pipes and the high biofilm formation potential of HDPE.

The members of *Proteobacteria* (*Alphaproteobacteria, Betaproteobacteria, Gammaproteobacteria, Deltaproteobacteria*) were identified in both the water and the biofilm samples from all pipes as could be expected on the basis of other drinking water systems studies (Vaz-Moreira et al. 2013; Kahlisch et al. 2010). The results of PCR-DGGE showed a smaller difference in diversity values, but higher in evenness indices between biofilm and the water sample in PVC pipes than in other pipes with extended biofilm. These tendencies could suggest that some species were strongly embedded in the biofilm structure and they occurred in water only in a small amount. It was opposite to results reported by Henne et al. (2012). They observed reduced richness of biofilm communities compared to bulk water. It could be a result of the presence of various residual DNA fragments in bulk water derived from surface water, especially that they observed completely different community composition in all bulk water samples determined by 16S rRNA gene analysis. In our studies the model water installation was supplied by a deep ground water originally nearly devoided of bacteria.

Various members of the order *Desulfomonadales* belonging to *Deltaproteobacteria* were reported to be involved in a reduction of variety of compounds including SO_4_(VI), Mn(IV), Fe(III) as electron acceptors in anaerobic respiration (Lovley et al. 2004). The abundance of these bacteria in the biofilm and water samples from HDPE pipes is probably related to the presence of large amounts of mineral deposits containing iron oxides precipitated on the pipe surface. An amplicon present only in the PEX biofilm but not found in the PEX water sample showed high similarity to *G. fermentans* (*Deltaproteobacteria*) that was able to reduce Fe(III). Nevin and Lovley (2002) have suggested that *G. fermentans* released an electron-shuttling compound that could transfer electrons from the cell to Fe(III) oxide that is not in contact with the organism. According to authors, bacteria release one or more compounds capable of chelating and solubilizing Fe(III) as a strategy for alleviating the need for a contact between cells and Fe(III) oxide for Fe(III) reduction. Therefore, *Geothrix* is able to take better advantage of high-redox-potential environments than other e(III)-reducing bacteria (Mehta-Kolte and Bond 2012). This could promote the appearance of these bacteria in the PEX biofilm that was rich in sediments containing iron oxides.

Extracellular polymers immobilize biofilm cells and protect them from biocides, xenobiotics and environmental factors: desiccation, UV irradiation, pH fluctuations, osmotic shock and shearing force. In drinking water systems and household installations exopolymer layer protects bacteria from disinfecting agents and other stressors (de Carvalho 2007) but it may also inhibit the access to nutrients. Norwood and Gilmour (2000) observed significant decrease of microbial concentrations in biofilm with 1,000 ppm of chlorine, while the effective chlorine concentration for disinfection of water was 10 ppm. In our study, *R. blasticus* and *W. maritima* belonging to *Alphaproteobacteria* were observed only in the biofilm from HDPE pipes. Mathieu et al. (2009) reported that *Alphaproteobacteria* have a higher sensitivity to high free residual chlorine concentrations (0.4 mg Cl_2_ L^−1^) than other classes of *Proteobacteria*. Presumably exopolymers in the extended biofilm on the HDPE surface may have protected these bacteria from the presence of the oxidant in water.

The most intensive DGGE bands (2, 4, 5) characterizing the water samples from the PEX lines corresponded to the DNA sequences of microorganisms belonging to *Sphingomonadales*, *Methylophilaceae* and *Acidobacteria*. Above mentioned bacteria are widespread in the environment due to their ability to utilize a wide range of organic compounds including refractory contaminants (Takeuchi et al. 1993; White et al. 1996). The microorganisms utilizing various C1-compounds such as formamide belong to the Family *Methylophilaceae* (Wyborn et al. 1994). In turn, the representatives of the order *Sphingomonadales* (i.g. *Sphingomonas*) may utilize more complex organic compounds eluted from plastic. Recently, Sun et al. (2013) identified *Sphingomonas* sp. resistant to 4 mg Cl_2_ L^−1^ in a model drinking water distribution system. Bacteria belonging to the phylum *Acidobacteria* are abundant in the environment, well suited to low-nutrient conditions and able to produce cellulose and excrete high-molecular-weight proteins that suggests their potential for desiccation resistance and biofilm formation. They were also detected in mature biofilm (1–3 years old) on stainless steel plugs in a model drinking water distribution system by Martiny et al. (2003). The growth of above mentioned groups of bacteria may suggest that some low-molecular-weight organics are released from PEX pipes.

## Conclusions

The results showed that biofilm formation potential of plastic pipes differs depending on the material which is used. The susceptibility of the tested materials to colonization and biofilm formation was: HDPE > PEX > PVC. 2 years of operation of three pilot installations in the same conditions resulted in the formation of biofilms that differed in structure and microbial composition on the inner surface of the pipes.

Although the diversity and the richness of the microbial communities was affected by the kind of pipe material used, the number of microorganisms on the inner surface of plastic pipes was not related to the abundance of mineral deposits. Biofilms in HDPE pipes were thickest but the total number of bacteria was the highest in PEX (1.24 × 10^7^ cm^−2^). Extended biofilms on HDPE pipes may be a source of potentially pathogenic and opportunistic bacteria such as *P. aeruginosa* or *Acinetobacter* sp. which were found on this material using culturable methods.
